# Are threat perceptions associated with patient adherence to antibiotics? Insights from a survey regarding antibiotics and antimicrobial resistance among the Singapore public

**DOI:** 10.1186/s12889-023-15184-y

**Published:** 2023-03-20

**Authors:** Si Yu Lee, Yang Shanshan, May O. Lwin

**Affiliations:** Wee Kim Wee School of Communication and Information, Nanyang Technological University, Singapore, Singapore

**Keywords:** Medication adherence, Protection motivation theory, Antibiotic knowledge, Antimicrobial resistance knowledge

## Abstract

**Background:**

Public health strategies to improve patient adherence to antibiotics rely mostly on raising awareness of the threat of antimicrobial resistance (AMR) and improving knowledge about antibiotics. We aimed to evaluate how adherence to antibiotics relates to knowledge and the threat perceptions proposed by the Protection Motivation Theory (PMT).

**Method:**

A cross-sectional online survey was conducted in September-December 2020 with 1002 participants aged 21-70 years in Singapore. Two items, which were reverse coded, evaluated adherence to antibiotics: ‘how often do you obtain antibiotics that were left over from the previous prescription’ and ‘how often did you treat yourself with antibiotics in the past year’. Questions about the PMT-related constructs, and knowledge regarding antibiotics and AMR knowledge were also included. Hierarchical regression models were performed at a 5% significance level.

**Results:**

Adherence to antibiotics was associated with knowledge level (β = 0.073, *p* < 0.05), education level (β = − 0.076, *p* < 0.01), and four of the five PMT constructs: “perceived response cost” (β = 0.61, *p* < 0.01), “perceived response efficacy of adherence to antibiotic” (β = 0.096, *p* < 0.01), “perceived susceptibility to AMR” (β = 0.097, *p* < 0.01), and “perceived severity of AMR” (β = − 0.069, *p* < 0.01). Knowledge about AMR, perceived self-efficacy in adhering to antibiotics, age, and sex were not associated with adherence.

**Conclusions:**

In Singapore, patient adherence to antibiotics appear to be driven by the perceived costs of visiting a doctor to obtain antibiotics, followed by perceptions of AMR as a threat and to a lesser extent, knowledge about antibiotics. Public health strategies to mitigate antibiotic misuse should consider these patient barriers to medical care.

**Supplementary Information:**

The online version contains supplementary material available at 10.1186/s12889-023-15184-y.

## Introduction

Antimicrobial resistance (AMR) is one of the top ten global public health threats [1]. While it is a naturally occurring process, the misuse and overuse of antimicrobials have accelerated AMR. In fact, a main cause of AMR is patient non-adherence to antibiotics [2], with a meta-analysis of studies conducted across four continents observing that nearly half of the respondents stopped their antibiotic course prematurely once they begin to feel better [3]. Adherence to antibiotics is, therefore, an important gap in the global efforts against AMR.

Patient adherence is a complex and multifactorial behavior that refers to the extent to which a patient’s behavior corresponds with recommendations from a healthcare provider [4, 5]. In the context of antibiotic use, adherence involves the patient completing the antibiotic course as prescribed and not self-medicating without a doctor’s prescription [6]. Patient-related determinants of suboptimal adherence include poor attitudes and perceptions toward antibiotics, poor understanding of the received regimen, and a rapid improvement in clinical outcomes [7–9]. On the clinician level, determinants include poor recognition of non-adherence, complex and multidrug prescriptions, and insufficient patient-physician communication [10]. On the health system level, determinants include poor inpatient and outpatient care coordination, and antibiotic accessibility [11]. This multiplicity of determinants makes the identification of modifiable determinants a critical research priority.

One major psychosocial determinant of adherence to antibiotics is how the public perceives the threat of AMR. Threat perceptions refer to how individuals appraise their susceptibility to and the severity of the adverse consequence of an event or action. As a core component of health behavioral theories such as the Protection Motivation Theory (PMT), threat perceptions have been identified as a driver of medication adherence in the management of chronic diseases such as diabetes and cardiovascular disease [12, 13]. However, no study to date has investigated how the public’s threat perceptions of AMR relate to their adherence to antibiotics.

This study aims to address this gap by applying the PMT to investigate the relationship between the public’s threat perceptions of AMR and their adherence to antibiotics. The PMT provides a theoretical framework to understand why and how people respond to health threats. It posits that people’s response to health threats depends on how they appraise the threats and the preventive action [14]. Then, according to PMT, the patient adherence to antibiotics will depend on their perceived severity of and vulnerability to AMR, as well as their perceived response efficacy, self-efficacy, and response cost of non-adherence.

This research is based in Singapore, a city-state in Southeast Asia with a population size of 5.5 million [15]. Antibiotics are largely classified as prescription-only drugs in Singapore under the regulation of the National Health Science Authority and it is usually dispensed to the public through primary care providers in government-funded polyclinics or private clinics operated by general practitioners [16]. The public’s knowledge of antibiotics and AMR are comparable to those of other countries - a recent study has found the public in Singapore to hold similar beliefs and misconceptions commonly identified in population-level antibiotic surveys of other countries [17].

## Material and methods

### Study design

The present study is performed as a part of a larger study to understand the knowledge, attitudes, and practices about antimicrobial use and AMR among the Singapore public. It was conducted from September to December 2020 using an online survey hosted on the survey platform Qualtrics. The survey respondents were recruited by Qualtrics following a set of criteria on age, gender, and ethnicity to ensure that the participant makeup is nationally representative. A total of 1002 citizens or permanent residents in Singapore between the ages of 21 and 70 were recruited for the study.

### Survey design

The survey was conducted in English which is commonly spoken in Singapore. To ensure validity, it was pilot tested to identify and rectify any issues. It has four main sections: (i) socio-demographic information, (ii) antibiotics-related behaviors, (iii) knowledge about antibiotics and AMR, as well as (iv) PMT-related perceptions about antibiotics and AMR. Relevant to the present study, socio-demographic information was measured using five questions on sex, residency status, ethnicity, education level, and monthly household income.

Patient Adherence to antibiotics was operationalized by measuring the dimensions related to use of leftover antibiotics and self-medication with antibiotics in the previous year.

The items are ‘how often do you obtain antibiotics that were leftover from the previous prescription’ and ‘how often did you treat yourself (self-medicate without a doctor’s prescription) with antibiotics in the past one year’. Participants answered these two questions on a five-point Likert scale (1 = ‘*never’* and 5 = ‘*always’*). Both items were then reverse coded and averaged into an adherence score that ranges from 1 (*low adherence*) to 5 (*high adherence*). The inter-item correlation of the 2 items is *r = 0.67.*

Knowledge about antibiotics was assessed using 28 true or false questions adapted from past studies [18, 19] (see TS 1). It comprises 11 questions on general facts about antibiotics, 10 questions on the diseases that can be treated with antibiotics, and 7 questions on the side effects of antibiotics. A score of one point was given for each correct answer. For each respondent, points were summated into an overall antibiotic knowledge score, which ranges from 0 to 28. Knowledge about AMR was assessed using 7 true or false questions adapted from the WHO’s multi-country public awareness survey on AMR [20] (see TS 2). One point was given for each correct answer and the points were then summated into an overall AMR knowledge score that ranges from 0 to 7 for each respondent.

Lastly, PMT-related perceptions of antibiotics and AMR were assessed with 17 questions using a 5-point Likert scale (See TS 3). An index for each of the PMT constructs was then created by summating the score for its constituting items.

### Data analysis

Descriptive analysis was run to assess the distribution of the participant’s socio-demographics, antibiotics knowledge, and AMR knowledge. Cronbach’s Alpha was also used to assess the internal consistency of adherence to antibiotics and the five constructs under PMT (see TS 4). All scales have a moderate to high level of reliability with a Cronbach’s alpha score of .66 to .81.

As guided by past applications of PMT in health [21, 22], we employed hierarchical regression to examine how adherence to antibiotic is associated with the PMT constructs, knowledge about antibiotics and AMR, as well as socio-demographical variables. This method allows us to compare the effect sizes across models comprising different predictors, and hence determine the best determinants of adherence to antibiotics. The hierarchical regression was conducted in a few steps. First, only age, sex, and education level were included to form regression Model 1. Then, knowledge about antibiotics and AMR were added to the socio-demographic variables to form regression Model 2. In the last step, we further added the five PMT constructs in the model to form regression Model 3. Additionally, we wanted to compare whether the composite constructs of threat appraisal (sum of perceived severity and perceived susceptibility) and coping appraisal (sum of perceived self-efficacy and perceived response efficacy minus perceived response cost) would be more effective than its five individual PMT constructs. We hence performed a separate regression model by replacing the five PMT constructs with threat appraisal and coping appraisal to form Model 4.

## Results

### Participants

The questionnaire was completed by 1002 participants. Thirty-five responses were removed as they were either completed in under 330 seconds or had duplicate IP addresses. The remaining 967 valid responses were used for analysis. Table 1 details the sociodemographic characteristics of the respondents. The average age of the respondents was 44.41 (*SD =* 12.14). The distribution of sex was roughly balanced with 50.8% female respondents and 49.1% male respondents. The ethnic distribution of the sample is also approximately representative of the ethnic makeup of the Singapore population in 2021 [23], with 75.0% of the respondents being Chinese, 14.5% being Malay, and 7.4% Indian.

**Table 1:** Sociodemographic characteristics of participants

***N*** = 967	
Sociodemographic Characteristics	n (%)
**Sex**	
Female	491 (50.8)
Male	475 (49.1)
**Residency Status**	
Singapore Citizen	846 (87.5)
Singapore Permanent Resident	121 (12.5)
**Age**	
21–29	130 (13.4)
30–39	234 (24.2)
40–49	257 (26.6)
50–59	223 (23.1)
60 and above	123 (12.7)
**Ethnicity**	
Chinese	725 (75.0)
Malay	140 (14.5)
Indian	72 (7.4)
Others	30 (3.1)
**Education**	
No formal schooling	4 (0.4)
PSLE or equivalent	9 (0.9)
GCE O/N Level	155 (16.0)
GCE A Level/ diploma	263 (27.2)
Degree/higher education	536 (55.4)
**Monthly household income (In SGD)**	
Below $1000	37 (3.8)
1000–4999	257 (26.6)
5000–9999	329 (34.0)
10,000–14,999	179 (18.5)
15,000–19,999	78 (8.1)
20,000 and over	61 (6.3)
Do not wish to answer	26 (2.7)

### Antibiotics adherence

Table 2 presents the descriptive statistics of respondents’ adherence to antibiotic recommendations. Adherence to antibiotics recommendations is generally high among the respondents with 60.8% of them “always adhering to antibiotics recommendations” (*N* = 578) and 26.8% adhering to them most of the time (*N* = 255).

**Table 2:** Descriptive statistics of adherence to antibiotics

	N	Mean (*SD*)	Frequency, N(%)				
			Never	Sometimes	Often	Most of the time	Always
Adherence to antibiotics	951	4.57 (0.73)	10 (1.1)	23 (2.4)	85 (8.9)	255 (26.8)	578 (60.8)
Item 1: Self-medicate without doctor’s prescription	953	1.45 (0.83)	670 (69.3)	183 (18.9)	64 (6.6)	23 (2.4)	13 (1.3)
Item 2: Use antibiotics from previous leftover prescription	965	1.41 (0.84)	714 (73.8)	162 (16.8)	48 (5.0)	24 (2.5)	17 (1.8)

### Knowledge about antibiotics and AMR

Respondents correctly answered an average of 12.5 (*SD* = 5.01) of the 28 questions on antibiotics. On the general statements asked about antibiotics, −respondents correctly answered an average of 3.61 (*SD* = 2.81) of the 11 questions. On the side effects of antibiotics, respondents correctly identified an average of 2.00 (*SD* = 1.33) of the 7 side effects of antibiotics, with fever, bloating, and vomiting being the three least known side effects. As for AMR knowledge, respondents correctly answered an average of 3.15 (*SD* = 1.71) of the 7 questions.

### Predicting adherence to antibiotics with PMT

Table 3 presents the descriptive statistics of the PMT constructs and their correlations. Of the five PMT constructs, perceived response cost has the lowest response rating with an average of 1.48 (*SD = .82)* on a 5-point Likert scale. This means that respondents, on average, tend to perceive adherence to antibiotic as an action with low cost. Perceived susceptibility to AMR has the second-lowest response rating with an average of 3.11 (*SD =* 0.77) on a 5-point Likert scale. This means that most respondents tended to be neutral about whether AMR will affect themselves or the people around them. Perceived response efficacy, on the other hand, has the highest response rating of 3.92 (*SD =* 0.74), meaning that respondents tended to believe that adherence to antibiotics is effective in coping with the threat of AMR.

**Table 3:** Descriptive statistics and intercorrelations of PMT constructs and adherence to antibiotics

Variables	Pearson correlation coefficient								Mean (*SD*)	Range
	1	2	3	4	5	6	7	8		
1. Adherence to antibiotics	–								4.57 (*0.73*)	1–5
2. Susceptibility	.27**	–							3.11 (*0.77*)	1–5
3. Severity	.08*	.005 (*p* = .12)	–						3.72 (*0.73*)	1–5
4. Self-Efficacy	.11**	−.07* (*p* = .03)	.49**	–					3.82 (*0.67*)	1–5
5. Response-Efficacy	.23**	0.04 (*p* = .21)	.53**	.48**	–				3.92 (*0.74*)	1–5
6. Response Cost	−.68**	−.28**	−.11**	−.10**	−.20**	–			1.49 (*0.82*)	1–5
7. Threat appraisal	.25**	.74**	.71**	.28**	.38**	−.28**	–		3.41 (*0.54*)	1–5
6. Coping appraisal	.51**	.13**	.53**	.70**	.78**	−.65**	.44**	–	2.08 (*0.52*)	1–5

*Denotes correlation is significant at the 0.05 level

**Denotes correlation is significant at the 0.01 level

The results also show that adherence to antibiotics is significantly correlated with all five PMT constructs. Coping and threat appraisals are significantly correlated with antibiotics adherence (*r* =. 51and .25 respectively, *p* < .01). Among the five constructs, the perceived response cost of adherence to antibiotics has the strongest correlation with adherence to antibiotics (*r =* −.69, *p* < .001). It is also the only construct negatively correlated with adherence to antibiotics. On the other hand, the perceived severity of AMR has the weakest correlation with adherence to antibiotics (*r =* .08, *p* = .01).

Table 4 shows the results of the hierarchical regression analysis to adherence to antibiotics based on the PMT model. **Model 1** served as the baseline that informs us how participants’ demographic traits could influence their adherence to antibiotics. It accounted for only 6.3% of the variance in adherence to antibiotics, *F* (3, 842) = 19.82, *p* < 0.001 with *R*^2^ = .066. For **Model 2**, the inclusion of the knowledge about antibiotics and AMR improved the effect size of the model, from *R*^2 =^ .066 to *R*^2^ = .14, *F* (2, 840) = 34.62, *p* < 0.001, specifically. Finally, **Model 3**, with five PMT constructs added to the analysis, presented the best predictive model with the biggest *R*^2^ change, *F* (5, 835) = 126, *p* < 0.001, which increased the *R*^2^ by .37. As shown in the results in Model 3, perceived response cost emerged as the most salient predictor of adherence to antibiotics, *t* = − 22.57, *p* < .001. One unit increase in perceived response cost decreases the average adherence score, which ranges from 1 to 5, by .61 ((*β =* .61, *p < 0.001)*. Perceived response efficacy of adherence to antibiotics (*t* = 3.1, *p* = .002; *β = .096)*, perceived susceptibility to AMR (*t* = 3.6, *p* < .001*; β = .097),* education level (*t* = − 3.02, *p* < .001, *β = −.076)*, antibiotic knowledge (*t* = 2.55, *p* = .01; *β = 0.073),* and perceived severity of AMR (*t* = − 2.17, *p* = .03, *β = −.069)* also emerged as significant predictors of adherence to antibiotics.

**Table 4:** Summary of hierarchical regression analysis for variables predicting adherence to antibiotics

Predictors	Model 1			Model 2			Model 3			Model 4		
	B	SE B	β	B	SE B	β	B	SE B	β	B	SE B	β
Age	0.013	0.002	0.222**	0.011	0.002	0.183**	0.003	0.002	0.049 (*p* = .06)	0.005	0.002	0.077* (*p* = .01)
Sex	0.043	0.049	0.029 (*p* = .38)	0.012	0.048	0.008 (*p* = .80)	−0.031	0.036	−0.021 (*p* = .40)	0.003	0.043	0.002 (*p* = .94)
Education level	−0.079	0.032	−0.084 (*p* = .01)	− 0.09	0.031	−0.095** (*p* = .004)	−0.072	0.024	−0.076** (*p* = .003)	−0.073	0.028	−0.078** (*p* = .009)
Antibiotics knowledge				0.044	0.005	0.287**	0.011	0.004	0.073* (*p* = .01)	0.016	0.005	0.104** (*p* = .002)
AMR knowledge				−0.033	0.015	−0.077* (*p* = .03)	−0.018	0.012	−0.041 *(p = .15)*	−0.064	0.014	−0.148**
Susceptibility							0.091	0.025	0.097**			
Severity							−0.070	0.032	−0.069** (*p = .03)*			
Self-Efficacy							0.041	0.033	0.036 (*p = .22)*			
Response-Efficacy							0.095	0.031	0.096** (*p = .002)*			
Response Cost							−0.544	0.024	−0.61**			
Threat appraisal										0.055	0.023	0.082* (*p* = .02)
Coping appraisal										0.212	0.016	0.455**
*R*^2^	0.066			0.137			0.508			0.316		
Adjusted *R*^2^	0.063			0.132			0.502			0.310		
F for change in *R*^2^	19.82**			34.62**			126.00**			109.22**		

*Denotes correlation is *p <* 0.05 level while ** denotes *p* < 0.01

In the last regression (**Model 4**), the five PMT dimensions were replaced with their overarching constructs – coping appraisal and threat appraisal. The three sociodemographic variables, two knowledge variables, together with the coping appraisal, and threat appraisal were entered together in the regression. The final model is also significant, *F* (7, 838) = 55.18, *p* < 0.01, However, it yields a smaller *R*^2^ change = .18 (*F* (2, 838) = 109.22, *p* < .001) as compared to that of model 3 (*R*^2^ change = .37) with the five dimensions of PMT included. Therefore, model 3 is the model with better predictive power.

## Discussion

This study aimed to investigate the utility of AMR threat perceptions in predicting and explaining adherence to antibiotics among the public. By applying the PMT to quantitatively explore this relationship, this research provides empirical evidence on AMR threat perceptions as modifiable psycho-social determinants of patient adherence to antibiotics. Our findings show that patient adherence to antibiotics can be significantly explained by PMT, with the five constructs accounting for 37.1% of the variance in this behavior. This amount of predictive variance exceeds those reported in past applications of PMT, such as in the study of breast cancer therapy adherence [24], preventative asthma treatment adherence [25], and eye patching adherence [26]. The PMT is henceforth a promising theoretical framework that can be used to understand why people fail to adhere to antibiotic recommendations and provide a blueprint for the development of future interventions.

Coping appraisal was found to exert a moderately-large effect on adherence to antibiotics with perceived response cost accounting for the largest effect among the five PMT constructs. This finding corroborates a meta-analysis of PMT studies which found response cost to be the most salient predictor of health behaviors [27]. The large negative effect size of perceived response cost suggests that one major determinant of non-adherence to antibiotics is the perception that people do not have the time and/or money to obtain antibiotics from healthcare providers and complete their prescribed antibiotic course. The smaller positive effect size of perceived response efficacy suggests that adherence is also, albeit to a notably smaller extent, driven by the belief that adhering to antibiotic recommendations can address the threat of AMR. Perceived self-efficacy, on the other hand, was not associated with adherence to antibiotics. This means that beliefs about whether an individual can enact behaviors to address AMR were not found to influence adherence to antibiotics. Collectively, these findings point to the utility of coping appraisal in predicting and explaining adherence to antibiotics with perceived response cost being the most promising modifiable psycho-social determinant.

We observed that the way individuals appraise the threat of AMR, as measured by perceived susceptibility and severity, has limited utility in explaining adherence to antibiotics, as very weak correlations were observed. This finding diverges from past literature which has found risk perceptions to be salient determinants of medication adherence for preventive or long-term treatment of chronic diseases [12, 13, 25]. One plausible reason for this contradictory finding is the psychological distance in which individuals view the risks of non-adherence to different medications. Psychological distance refers to how removed an event is perceived to be from direct experience [28]. Heightened psychological distance has been shown to reduce people’s intentions to adopt behaviors in a wide variety of contexts such as climate change [29] and the consumption of sugar-sweetened beverages [30]. While psychological distance has yet to be examined in the context of medication adherence, it is tenable that people are more likely to perceive the risks of antibiotics non-adherence, which are often less direct, immediate, and salient than those associated with other medication, as more psychologically distant. According to the construal level theory, this increased psychological distance can, in turn, reduce the effect of threat perceptions in motivating individuals to adhere to antibiotic recommendations [31]. Future studies could hence explore the saliency of psychological distance as a possible psycho-social barrier to adherence to antibiotics as well as examine the interactions between psychological distance and AMR threats perceptions on adherence. Future public health interventions can also explore ways to reduce people’s perceived psychological distance to AMR as a way to improve patient adherence to antibiotics.

On the other hand, while knowledge about antibiotics and knowledge regarding AMR were indeed found to be statistically significant determinants of adherence, they only accounted for 6.9% of its variability. The magnitude of the effect of knowledge on adherence was even more reduced when the PMT constructs were considered. This finding offers some viable explanations for the mounting body of conflicting evidence between knowledge and adherence to antibiotics [32]. While some studies have found higher levels of antibiotic knowledge to be associated with lower levels of adherence, others have found support for the reverse, and some even found the lack of relationship between antibiotic knowledge and adherence [33].

### Study limitations

The results from our study should be considered in light of several potential limitations that future research could address. First, this was a single-country study conducted in Singapore, a developed nation in Southeast Asia, and our findings may be less generalizable to nations with different economic, cultural, and development profiles. Second, the self-report of adherence is prone to desirability and recall biases that can underestimate the prevalence of adherence. Besides, as the general public may not be able to fully distinguish antibiotics from other medications, it cannot be ruled out that our measure of adherence to antibiotics captures how well respondents adhere to medication in general instead of antibiotics specifically. However, as we have emphasized in the survey that this was a study on antibiotics, we believe that the two items we have chosen are with sufficient face validity. Since non-adherence to antibiotics includes various types of behaviors, future studies should include other dimensions such as not being compliant with the intake schedule. Third, participants may have used online search tools when completing the self-administered survey – a limitation that can overestimate the knowledge level regarding antibiotics and AMR.

### Insights for future antibiotic stewardship programs

Our findings may translate into practical implications for future antibiotic stewardship programs, suggesting other targets besides the lack of understanding of responsible antibiotics practices or adverse consequences of AMR. In fact, the large effect of coping appraisal and the comparatively weaker effect of threat appraisals suggest that persuasion strategies aimed at heightening concerns about AMR, such as the use of fear appeals, might not be as effective, and that may be better served with constructive communication strategies which include health education. In addition, future interventions should consider ways to assuage the public’s concerns about costs and time needed to adhere to antibiotic recommendations.

### Conclusion

As a pioneering study to examine how AMR threat perceptions relate to patient adherence to antibiotics, we found that while adherence was indeed driven by people’s perceptions of the severity of and their susceptibility to AMR, the effect of AMR threat perceptions was very weak. Adherence was instead found to be more influenced by how people perceived antibiotic-related costs. Knowledge about AMR and antibiotics was found to exert very little effect on adherence. Taken together, these findings demonstrate the complex and multifactorial nature of patient adherence to antibiotics and the relevancy of psychosocial constructs to the design of public health interventions.

## Supplementary Information

**Additional file 1.**

## Data Availability

The data is available upon request to the corresponding author.
