# Supplementary material for: Are threat perceptions associated with patient adherence to antibiotics? Insights from a survey regarding antibiotics and antimicrobial resistance among the Singapore public
Source: BMC Public Health. 2023 Mar 20;23:532. doi: 10.1186/s12889-023-15184-y (PMC10029286; doi:10.1186/s12889-023-15184-y)
Supplement: Supplementary file 1 — Additional file 1. [file 12889_2023_15184_MOESM1_ESM.docx]

**Supplemental Tables**

**TS 1**

| List of Antibiotics Knowledge Items | | T/F |
| --- | --- | --- |
| **General statements about antibiotics** | |  |
|  | Antibiotics are medicines that can treat bacterial infections. | T |
|  | If you take the same antibiotics repeatedly, they could stop working. | T |
|  | Antibiotics and anti-inflammatory drugs are the same drug. | F |
|  | The more expensive the antibiotic, the more effective it will be. | F |
|  | Antibiotics are medicines that can treat fungal infections. | T |
|  | Administration of multiple antibiotics has better efficacy than that of single one. | F |
|  | You always need antibiotics if your phlegm is green. | F |
|  | Switching to different antibiotics reduces side effects of antibiotics. | F |
|  | Antibiotics are medicines that can treat viral infections. | F |
|  | Most cold, cough and flu illnesses get better faster with antibiotics. | F |
|  | Scientists can always produce new antibiotics. | F |
| **Diseases that can be treated with antibiotics** | |  |
|  | Runny nose | F |
|  | Aches and pains | F |
|  | Diarrhea | F |
|  | Vomiting | F |
|  | Cough | F |
|  | Flu | F |
|  | Pneumonia | T |
|  | Skin or wound infection | T |
|  | Fever | F |
|  | Sore throat | F |
| **Side effects of antibiotics** | |  |
|  | Kill healthy bacteria | T |
|  | Rash | T |
|  | Diarrhea | T |
|  | Loss of appetite | T |
|  | Vomiting | T |
|  | Bloating | T |
|  | Fever | T |

**TS 2**

| List of Antimicrobial Resistance Knowledge Items | T/F |
| --- | --- |
| Antibiotic resistance occurs when your body becomes resistant to antibiotics, and they no longer work as well. | F |
| Many infections are becoming increasingly resistant to treatment by antibiotics. | T |
| Antibiotics are widely used in agriculture (including in food producing animals). | T |
| Resistant bacteria arising in one country cannot spread to other countries. | F |
| Bacteria which are resistant to antibiotics can be spread from person to person. | T |
| Resistant bacteria arising in humans, animals or the environment cannot spread from one to the other. | F |
| Antibiotics use in ensuring health livestock and promoting animal growth do not cause antibiotic resistance. | F |

**TS 3**

| List of items under the Protection Motivation Theory | |
| --- | --- |
| **Perceived susceptibility to AMR** | |
|  | 1. Antibiotic resistance is an issue in other countries but not in Singapore. |
|  | 1. Antibiotic resistance is only a problem for people who take antibiotics regularly. |
|  | 1. Antibiotic resistance is not an issue that could affect me or my family. |
|  | 1. I am not at risk of getting an antibiotic-resistant infection, as long as I take my antibiotics correctly. |
| **Perceived severity of AMR** | |
|  | 1. It is dangerous to people if pathogens become resistant to antibiotics. |
|  | 1. if bacteria are resistant to antibiotics, it can be very difficult or impossible to treat the infections they cause. |
|  | 1. Antibiotic resistance is one of the biggest problems the world faces. |
| **Perceived self-efficacy of antibiotic adherence** | |
|  | 1. There's not much I can do to stop antibiotic resistance (reverse coded). |
|  | 1. Everyone needs to take responsibility for using antibiotics responsibly. |
|  | 1. Most people including myself can help keep antibiotic resistance under control. |
|  | 1. I can take actions to prevent antibiotic resistance. |
| **Perceived response efficacy of antibiotic adherence** | |
|  | In response to ‘How much do you agree or disagree that the following actions would help address the problem of antibiotic resistance?’ |
|  | 1. People should use antibiotics only when they are prescribed by a doctor or nurse. |
|  | 1. People should not keep antibiotics and use them later for other illnesses |
| **Perceived response cost of antibiotic adherence** | |
|  | 1. I did not have enough time to visit a doctor. |
|  | 1. I did not have enough money to pay the clinic or hospital visit. |

**TS 4**

| Cronbach's Alpha and Mean Inter item Correlation (MIIC) | | | |
| --- | --- | --- | --- |
|  |  | Cronbach's α | MIIC |
| Antibiotic Adherence | | 0.67 | 0.51 |
| Protection Motivation Theory | |  |  |
|  | Perceived Susceptibility | 0.75 | 0.55 |
|  | Perceived Severity | 0.80 | 0.65 |
|  | Perceived Response Efficacy | 0.66 | 0.47 |
|  | Perceived Self-efficacy | 0.74 | 0.57 |
|  | Perceived Response Cost | 0.81 | 0.68 |
